# Differential responsiveness to immunoablative therapy in refractory rheumatoid arthritis is associated with level and avidity of anti-cyclic citrullinated protein autoantibodies: a case study

**DOI:** 10.1186/ar2309

**Published:** 2007-10-10

**Authors:** YK Onno Teng, Robert J Verburg, Kirsten N Verpoort, Gwendolyn MP Diepenhorst, Ingeborg M Bajema, Maarten JD van Tol, Els CM Jol-van der Zijde, Rene EM Toes, Tom WJ Huizinga, Jacob M van Laar

**Affiliations:** 1 Department of Rheumatology, Leiden University Medical Center, Albinusdreef 2, 2333 ZA Leiden, The Netherlands; 2 Department of Immunopathology, Sanquin Research, Academic Medical Centre, Plesmanlaan 125, 1066 CX Amsterdam, The Netherlands; 3 Department of Pathology, Leiden University Medical Center, Albinusdreef 2, 2333 ZA Leiden, The Netherlands; 4 Department of Pediatrics, Leiden University Medical Center, Albinusdreef 2, 2333 ZA Leiden, The Netherlands; 5 Musculoskeletal Research Group, Institute of Cellular Medicine, School of Clinical Medical Sciences, Newcastle University, 4th Floor, Catherine Cookson Building, The Medical School, Framlington Place, Newcastle upon Tyne, NE2 4HH, United Kingdom

## Abstract

In order to identify pathogenic correlates of refractory rheumatoid arthritis (RA), antibodies against anti-cyclic citrullinated protein (ACPAs) were investigated in RA patients in whom the dysregulated immune system had been ablated by high-dose chemotherapy (HDC) and autologous haematopoietic stem cell transplantation (HSCT). Six patients with refractory RA were extensively characterized in terms of levels of total immunoglobulins, RA-specific autoantibodies (ACPAs and rheumatoid factor) and antibodies against rubella, tetanus toxoid (TT) and phosphorylcholine before and after HDC plus HSCT. Additionally, the avidity of ACPAs was measured before and after treatment and compared with the avidity of TT antibodies following repeated immunizations. Synovial biopsies were obtained by arthroscopy before HDC plus HSCT, and analyzed by immunohistochemistry. In the three patients with clinically long-lasting responses to HDC plus HSCT (median 423 days), significant reductions in ACPA-IgG levels after therapy were observed (median level dropped from 215 to 34 arbitrary units/ml; *P *= 0.05). In contrast, stable ACPA-IgG levels were observed in three patients who relapsed shortly after HDC plus HSCT (median of 67 days). Clinical responders had ACPA-IgG of lower avidity (*r *= 0.75; *P *= 0.08) and higher degree of inflammation histologically (*r *= 0.73; *P *= 0.09). Relapse (after 38 to 530 days) in all patients was preceded by rising levels of low avidity ACPA-IgG (after 30 to 388 days), in contrast to the stable titres of high avidity TT antibodies. In conclusion, humoral autoimmune responses were differentially modulated by immunoablative therapy in patients with synovial inflammation and low avidity ACPA-IgG autoantibodies as compared with patients with high levels of high avidity ACPA-IgG. The distinct clinical disease course after immunoablative therapy based on levels and avidity of ACPA-IgG indicates that refractory RA is not a single disease entity.

## Introduction

Rheumatoid arthritis (RA) is a systemic, chronic and progressive disease that requires long-term immunosuppressive treatment, in which disease-modifying antirheumatic drugs (DMARDs) play a central role. However, several studies have shown that failure rates with conventional DMARD therapy can reach 75% over a follow-up period of 5 years [1-3]. High-dose chemotherapy (HDC) followed by autologous haematopoietic stem cell transplantation (HSCT) is employed in the treatment of patients with refractory autoimmune diseases, including systemic lupus erythematosus (SLE), systemic sclerosis and RA [4]. However, clinical efficacy of HDC plus HSCT varies between different autoimmune diseases. A recent review of the European Group for Blood and Marrow Transplantation/European League Against Rheumatism registry for autologous HSCT in autoimmune disease [5] showed that sustained improvements were common in patients with systemic sclerosis and systemic lupus erythematosus, whereas in RA temporary improvements with subsequently relapsing disease was the most common clinical course. Although the therapeutic mechanism of HDC plus HSCT is conceptually similar for all autoimmune diseases, it is currently unclear why HDC plus HSCT exhibited inferior efficacy in RA.

A common finding in autoimmune diseases is activation of autoreactive B lymphocytes, resulting in the formation of disease-specific autoantibodies [6,7]. Although the contribution of autoantibodies to the pathogenesis of autoimmune diseases is still unclear, many studies have demonstrated that the presence of autoantibodies has diagnostic significance [8-10] and is associated with worse disease outcome [11-14]. In RA the presence of IgM rheumatoid factor (RF) and anti-cyclic citrullinated protein antibody (ACPA)-IgG can be demonstrated years before the clinical onset of RA [15], indicating that humoral autoimmunity had been elicited before the development of overt autoimmune disease. Additionally, their presence was associated with disease progression [16] and the levels of ACPA-IgG predicted responsiveness to antirheumatic drugs [17]. However, the precise mechanisms underlying the humoral autoimmune response in RA patients are still poorly defined [18]. The majority of studies on ACPA-IgG have investigated ACPA-IgG responses at a time when overt autoimmune disease was already established. In these studies, treatment with conventional immunosuppressive drugs or biological agents did not result in the elimination of circulating autoantibodies [19]. The latter finding has been attributed to the persistence of autoreactive, memory T and B lymphocytes, the existence of long-lived autoreactive plasma cells [20,21], or repeated activation and differentiation of new autoreactive lymphocytes [22,23].

The present study exploited the profound anti-inflammatory, anti-proliferative, and immunoablative effects of HDC plus HSCT [24,25] to investigate whether humoral autoimmune responses to ACPAs can be abrogated in refractory RA and whether relapses are accompanied by newly generated autoimmune responses.

## Materials and methods

### Patients and sample collection

Six patients with severe RA treated with HDC plus HSCT were included in the study. From the original study cohort of 14 patients [26], eight patients were treated and extensively followed up at Leiden University Medical Center. The present study involves the six patients who were seropositive for RF-IgM as well as ACPA-IgG, and for whom extensive clinical data and experimental data were available. All patients had an established diagnosis of RA based on American College of Rheumatology criteria [27] with progressive erosive disease, including large joint involvement, and were refractory to combination therapy with DMARDs and, in four patients, with tumour necrosis factor (TNF)-blocking agents. Heparinized whole blood was collected and peripheral blood mononuclear cells were isolated by density gradient centrifugation over Ficoll-amidotrizoate (Leiden University Medical Center, Leiden, The Netherlands) and frozen in liquid nitrogen until analysis. Peripheral blood mononuclear cells were collected every 3 months after treatment. Serum was collected and stored at -20°C every month during the first year after treatment and every 3 months during the second year after treatment. The protocol was approved by the Leiden University Medical Center Ethics Committee and all patients provided written informed consent.

### Immunological monitoring and clinical evaluations

RA disease activity was assessed by the Disease Activity Score for 44 joints (DAS_44_) [28]. To correlate the results of immunological monitoring with individual disease courses, patients' clinical follow up was split in four consecutive periods, marked by the date of HSCT and by the first symptoms of relapse after HSCT. Thus, four distinct periods were defined: before HSCT, after HSCT ('nadir'), DMARD-free period after HSCT ('DMARD-free') and after clinical relapse after transplantation ('relapse'). Relapse was independently assessed by two rheumatologists and necessitated reinstitution of conventional antirheumatic treatment.

### Immunohistochemical analysis

Synovial tissue specimens were obtained during arthroscopy, which was performed before treatment on clinically affected knees, as described previously [29]. At each procedure 16 to 20 pieces of synovial tissue were collected using 2.0 mm grasping forceps (Storz, Tuttlingen, Germany) and embedded in paraffin until analysis. Paraffin-embedded, serial sections were stained with the following antibodies: rabbit anti-huCD3 (clone-SP7; Neomarkers, Fremont, CA, USA), mouse anti-huCD79a (clone-JCD117; Dako, Glostrup, Denmark), mouse anti-huCD20cy (clone-L26; Dako), mouse anti-human Ki-67 (clone-MIB-1; Dako), mouse anti-huCD68 (clone-KP1; Dako) and mouse anti-huCD138 (clone B-B4, Serotec, Dusseldorf, Germany). Sections were de-paraffinized by xylol, ethanol and demi-water, followed by antigen retrieval via 10 minutes of incubation of sections in boiling 1 mmol/l EDTA or 10 mmol/l citrate buffer. After washing in demi-water and phosphate-buffered saline, the appropriate titrated amount of antibody was added and incubated for 60 minutes at room temperature. Thereafter, sections were incubated with Mouse Envision or Rabbit Envision conjugate (Dako) for 30 minutes. The colouring reaction was completed with DAB substrate (Dako). Counterstaining was performed with haematoxylin. Sections were covered with Micromount mounting medium (Surgipath, Peterborough, UK).

### Semiquantitative scoring of inflammation

Stained sections were coded and randomly analyzed. All areas of each biopsy section were scored blindly by two independent observers. Stained sections were scored semi-quantitatively from 0 to 4 (1 = lowest and 4 = highest level of expression) [30]. The scoring system was calibrated for each marker separately. Inflammation scores (ranging from 0 to 16) were determined by the sum of four components: thickness score for synovial lining (as scored by the number of cells constituting the synovial lining) and semiquantitative scores (ranging from 0 to 4) for polymorphonuclear cells, lymphocytes and plasma cells [31]. Differences between observers were resolved by mutual agreement.

### Serum antibody levels

Serial serum samples were analyzed for levels of total immunoglobulins, RA-specific autoantibodies and physiological antibodies. Total serum IgG and IgM levels were measured by immunoturbidimetry using the COBAS-Integra 400/700/800 (Roche Diagnostics, Indianapolis, IA, USA), in accordance with the manufacturer's guidelines.

A commercial ELISA was used to measure serum antibodies against cyclic citrullinated peptides (Immunoscan RA, mark 2; Euro-Diagnostica, Arnhem, The Netherlands), in accordance with the manufacturer's instructions. Consistent with the international literature, we use the general term 'ACPA-IgG' to refer to the entire family of autoantibodies to citrullinated proteins of the IgG isotype. Serum RF-IgM was measured using a standardized ELISA [32]. Serum levels of rubella of the IgG isotype (RL-IgG) were measured using the MEIA, Axsym^® ^System (Abbott Diagnostics, Abbott Park, IL, USA), in accordance with the manufacturer's recommendations. RL-IgG levels were considered positive when they were equal to or above 10 IU/ml. Serum levels of anti-phosphorylcholine of the IgM isotype (PC-IgM) were measured using ELISA, as described previously [33]. Dilutions of a serum pool, obtained from 200 healthy volunteers, were used as standard, which was arbitrarily set at 100 units/ml of PC-IgM antibodies. Results were related to this standard and expressed as arbitrary units (AU)/ml.

### Tetanus toxoid immunizations

Patients were given repeated immunizations with tetanus toxoid (TT), which was part of the DTPol vaccine (National Institute of Public Health and Environmental Protection, Bilthoven, The Netherlands), containing diphtheria toxoid, tetanus toxoid (five flocculation units), and inactivated poliovirus types 1, 2 and 3. Patients were boosted at 3, 4 and 5 months after HDC plus HSCT when leucocyte counts had normalized. During the period of immunizations, two patients (patients 4 and 5) had restarted DMARD treatment because of a relapse of RA. Serum was collected before each immunization and approximately 1 month after the last immunization and stored at -20°C.

### Avidity measurements

Avidity of ACPA-IgG and TT-IgG was measured by a modified elution ELISA, as previously described [34]. Briefly, dilutions of serum samples were allowed to interact with cyclic citrullinated peptide or TT coated in the wells of microtitre plates. After 1 hour incubation of 100 μl of diluted serum samples per well, ELISA plates were washed. Thereafter, wells were incubated with 150 μl of a variable molarity (range 0.5 to 6.0 mol/l) of the chaotropic agent sodium thiocyanate (NaSCN; Merck, Darmstadt, Germany) for 15 minutes at room temperature. After vigorous washing, conjugate and colour reactions were performed according to the corresponding ELISA described above. The relative avidity index was defined as the molarity of NaSCN at which 50% of the amount of IgG antibodies bound to the coated antigen in the absence of NaSCN had been eluted from the antigen.

### Statistical analysis

Levels of autoantibodies, antibodies and immunohistochemical scores within patients were compared with their corresponding pre-transplantation values by using the nonparametric Wilcoxon signed rank test. Correlations between synovial inflammation and ACPA-IgG level and ACPA-IgG avidity, as well as correlations between ACPA-IgG levels and C-reactive protein levels were tested using linear regression analysis. Tests were considered significant at *P *≤ 0.05.

## Results

### Patients

As summarized in Table 1, all patients had persistent RA with a median disease duration of 12 years (range 7 to 20 years) and were positive for RF-IgM and ACPA-IgG, with a pre-transplantation median RF-IgM level of 836 IU/ml (range 61 to 1,796 IU/ml) and ACPA-IgG level of 484 AU/ml (range 94 to 1,584 AU/ml). All patients were heterozygous for shared epitope alleles. Patients had high disease activity before transplantation, as indicated by a median DAS_44 _score of 5.31 (range 4.58 to 7.24). After treatment with HDC plus HSCT all patients exhibited an improvement in clinical disease activity, with a lowest median DAS_44 _of score 1.95 (range 0.89 to 3.80). Importantly, in two patients (patients 4 and 6) the DAS_44 _score did not decrease to below 2.4 (cut-off for active disease according to European League Against Rheumatism response criteria [35]). The improvements in DAS_44 _score were temporary, and patients were off DMARD therapy for a median duration of 174 days (range 60 to 738 days). At the time of relapse, when DMARDs were reinstituted, the median DAS_44 _score was 3.91 (range 2.62 to 5.16).

**Table 1:** Overview of study patients

	Pre-transplantation characteristics								Post-transplantation characteristics		
	
	Age (years)	Sex	Disease duration (years)	RF-IgM (IU/ml)	ACPA-IgG (AU/ml)	CRP (mg/l)	ESR (mm/hour)	DAS_44_	Lowest DAS_44_	DMARD-free days	DAS_44 _at start or DMARDs
Patient 1	51	Female	7	575	94	12	40	5.89	0.89	423	2.62
Patient 2	43	Male	11	1,096	215	57	126	5.13	1.95	163	4.10
Patient 3	42	Female	15	500	401	50	61	4.58	1.94	738	3.40
Patient 4	35	Female	9	61	577	2	49	4.77	3.52	60	5.16
Patient 5	45	Female	12	1,656	1,014	192	113	5.49	1.82	67	3.72
Patient 6	42	Female	20	1,796	1,584	59	64	7.24	3.80	184	4.95

ESR, erythrocyte sedimentation rate; DAS_44_, Disease Activity Score of 44 joints; DMARD, disease-modifying antirheumatic drug.

### Immunoablative therapy resulted in selective reduction of serum ACPA-IgG

**Figure 1:**
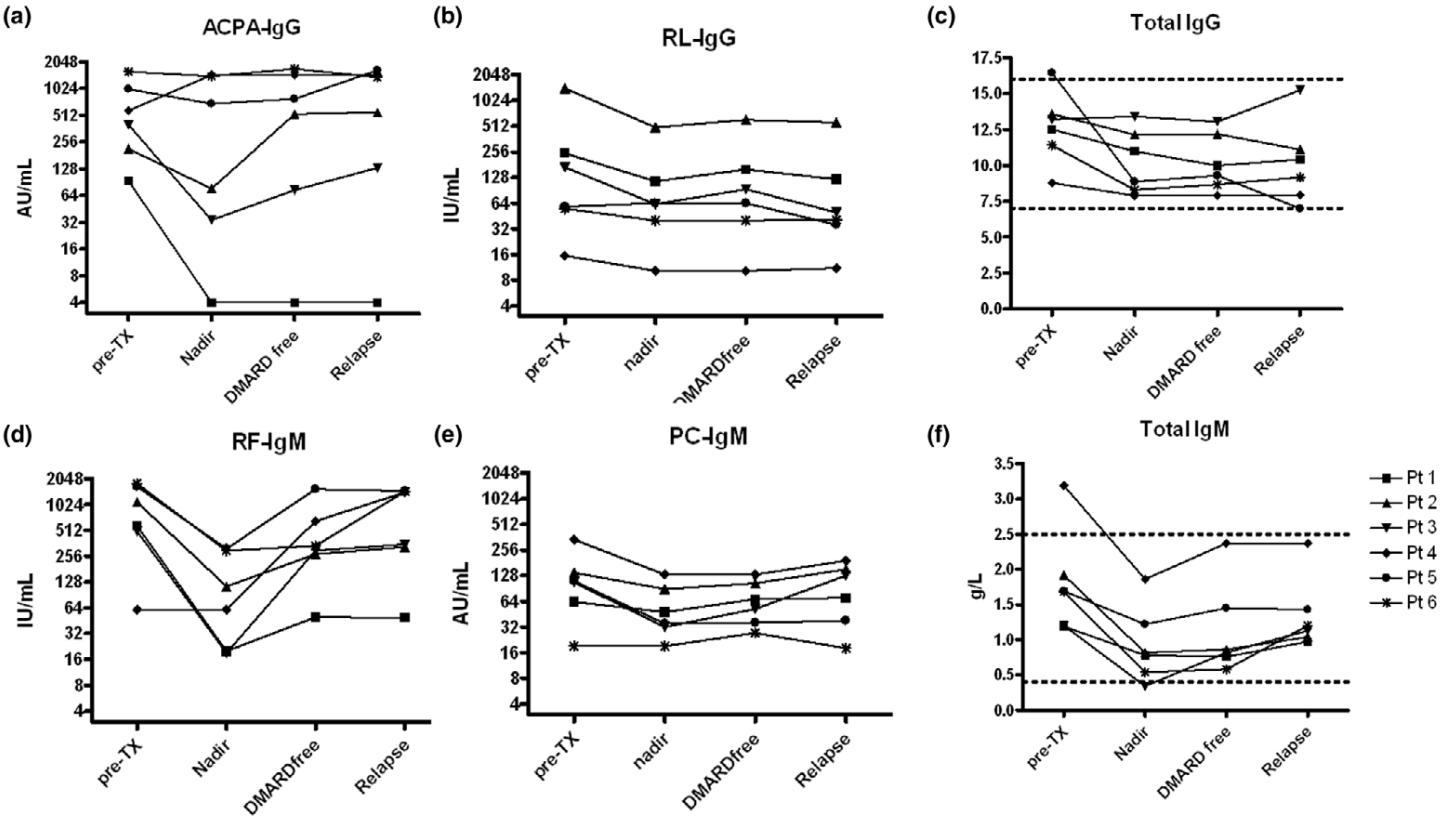
Effects of immunoablative therapy. Shown are the effects of immunoablative therapy on rheumatoid arthritis (RA)-specific autoantibody responses, physiological antibody responses and total immunoglobulin levels. The individual disease course of patients was split in four consecutive time periods: before immunoablative therapy ('pre-TX'), the lowest antibody level after immunoablative therapy ('nadir'), the period after immunoablative therapy without disease-modifying antirheumatic drug (DMARD) therapy ('DMARD free'), and the time of overt clinical flare of synovitis ('relapse'). Because the duration of the DMARD-free period varied for each patient (see Table 1), the presented levels are a mean of all observations during that period. Each symbol corresponds to a different patient. **(a) **Titers of antibodies against anti-cyclic citrullinated protein (ACPA)-IgG. The cut-off for ACPA-IgG is 25 arbitrary units (AU)/ml. In patient 1 a long-lasting undetectable level of ACPA-IgG was observed after high-dose chemotherapy plus haematopoietic stem cell transplantation. Levels below the detection limit were arbitrarily assigned a value of 4 AU/mL to optimize the graphical representation. **(b) **Titres of rubella of the IgG isotype (RL-IgG). **(c) **Titres of total circulating IgG. **(d) **Titres of rheumatoid factor (RF)-IgM.**(e) **Titres of anti-phosphorylcholine of the IgM isotype (PC-IgM). **(f) **Titres of total circulating IgM.

Although clinical responses to HDC plus HSCT were heterogeneous, immunoablative therapy resulted consistently in complete depletion of circulating B and T lymphocytes, and in significant reductions of synovial inflammation (data previously reported [26]). To investigate whether clinical responses were associated with eradication of autoantibodies, we analyzed the influence of immunoablative therapy on the levels of ACPA-IgG and RF-IgM in comparison with antibody levels against control antigens, respectively RL-IgG and PC-IgM (selected because of the high prevalence of seropositivity in the Dutch population), and the total IgG and total IgM levels (Figure 1).

In three patients (patients 1 to 3) pre-transplantation levels of ACPA-IgG (median 215 AU/ml, range 94 to 401 AU/ml) were nearly completely eradicated after HDC plus HSCT (nadir median 34 AU/ml, range <20 to 77 AU/ml; *P *= 0.05). In one of these patients (patient 2) ACPA-IgG remained undetectable for the duration of follow up (757 days; Figure 1a). These decreases contrasted with the stable levels of the RL-IgG control antibody in all patients (median pre-transplantation level 114 IU/ml [range 16 to 1,430 IU/ml] versus median nadir level 63 IU/ml [range 10 to 494 IU/ml], *P *= 0.63; Figure 1b). Immunoablative therapy also resulted in significant reductions in both RF-IgM levels (median pre-transplantation level 1,125 IU/ml [range 500 to 1,796 IU/ml] versus median nadir level 344 IU/ml [range 19 to 1,210 IU/ml, *P *= 0.043; Figure 1d) and PC-IgM levels (median pre-transplantation level 109 AU/ml [range 19 to 340 AU/ml] and median nadir level 41 AU/ml [range 19 to 132 AU/ml, *P *= 0.043; Figure 1e) in the entire cohort. Importantly, serum levels of total IgM and IgG were also affected by HDC plus HSCT, notably IgM (Figure 1c,f). In individual patients, the relative reduction in RF-IgM paralleled the relative reduction in total IgM (median correlation coefficient 0.78, range 0.34 0.95), whereas the relative reduction in ACPA-IgG was only weakly correlated with the relative reductions in total IgG levels (median correlation coefficient 0.33, range 0.08 to 0.81; *P *= 0.07), which is indicative of nonselective depletion of RF-IgM.

### Synovial inflammation is accompanied by ACPA-IgG autoantibodies of low avidity

In the patients exhibiting a significant drop in ACPA-IgG (patients 1 to 3) we observed a more pronounced decrease in DAS_44 _scores (0.89 to 1.95 versus 1.82 to 3.80; *P *= 0.27) and more DMARD-free days (163 to 738 days versus 60 to 184 days, *P *= 0.12) after HDC plus HSCT as compared with the three patients with stable ACPA-IgG levels (patients 4 to 6; Table 1). Therefore, we explored the possibility that persisting synovial inflammation, as a putative source of ACPA-IgG production, was associated with high and stable ACPA-IgG levels.

**Figure 2:**
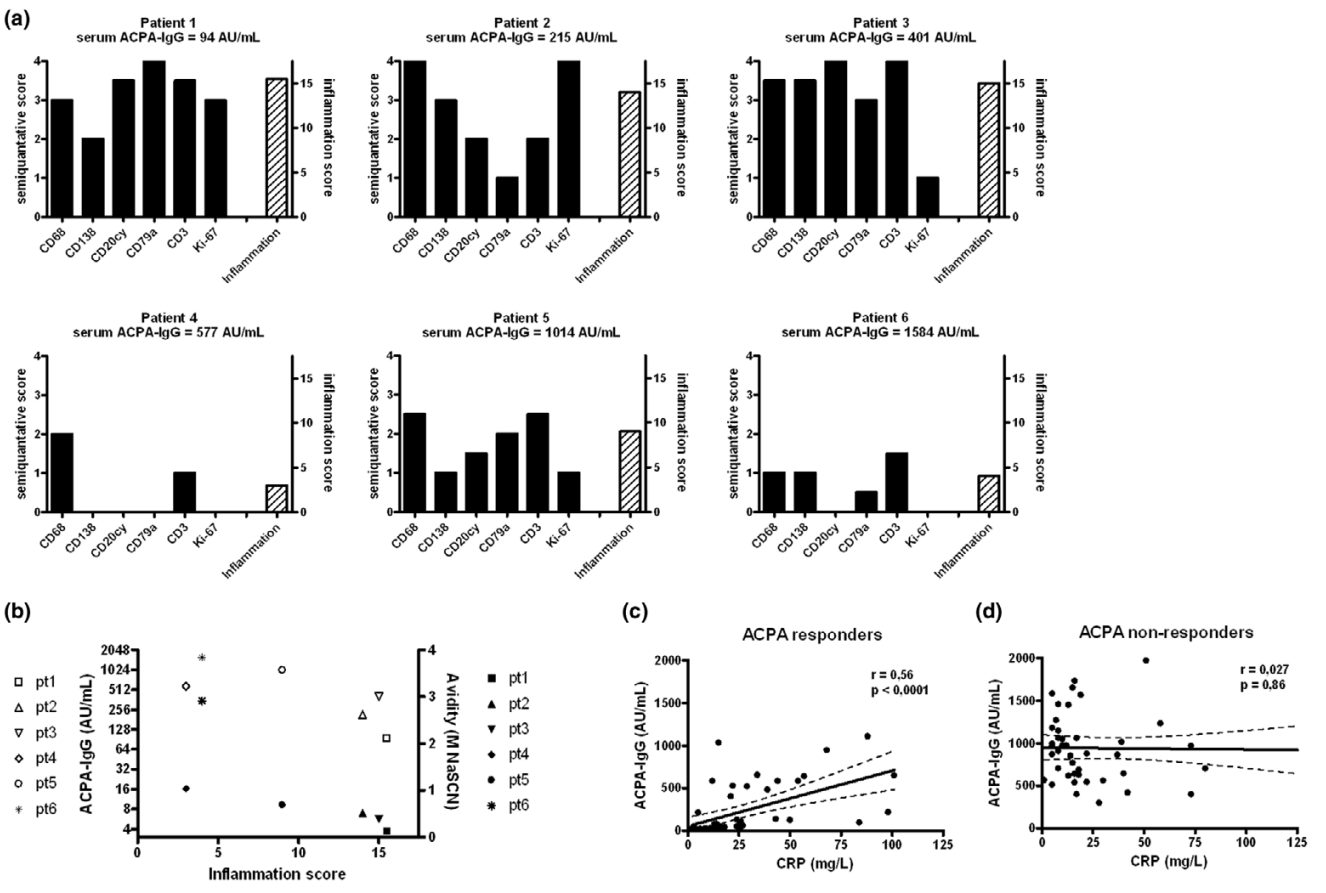
Associations between synovial inflammation and circulating ACPA-IgG autoantibodies. **(a) **Semiquantitative scores of individual patients before immunoablative therapy, scoring synovial infiltration for CD68 expression (macrophages), CD138 expression (plasma cells), CD20 expression, CD79a expression (B cells), CD3 expression (T-cells), Ki-67 expression (proliferation marker) and total inflammation. **(b) **Correlation of synovial inflammation scores with serum levels of antibodies against anti-cyclic citrullinated protein (ACPA)-IgG (open symbols) and avidity of ACPA-IgG (closed symbols) before immunoablative treatment. Each symbol corresponds to a distinct patient. **(c) **Correlation of serum levels of ACPA-IgG with C-reactive protein (CRP) levels (*r *= 0.56, *P *< 0.001) during complete follow up in the three patients whose ACPA-IgG levels are susceptible to immunoablative therapy ('ACPA responders'). **(d) **Correlation of serum levels of ACPA-IgG with CRP levels (*r *= 0.027, *P *= 0.86) during complete follow up in the three patients whose ACPA-IgG levels remained stable after immunoablative therapy ('ACPA nonresponders'). AU, arbitrary units; NaSCN, sodium thiocyanate.

Cross-sectional analysis of pre-transplantation synovial tissue specimens revealed a heterogeneous degree of inflammation involving various cell types (Figure 2a). Unexpectedly, a high degree of synovial inflammation was correlated with relatively low ACPA-IgG levels (*r *= 0.73, *P *= 0.09) and relatively low avidity of ACPA-IgG (*r *= 0.75, *P *= 0.08; Figure 2b). These findings were corroborated by the observation that, throughout the complete study period, ACPA-IgG levels were strongly correlated with serum C-reactive protein levels in the three patients (patients 1 to 3) whose ACPA-IgG levels were nearly completely eradicated after immunoablative therapy ('ACPA responders': *r *= 0.56, *P *< 0.001; Figure 2c). The latter was not the case for the three patients (patients 4 to 6) whose ACPA-IgG remained relatively stable after immunoablative therapy ('ACPA nonresponders': *r *= 0.027, *P *= 0.86; Figure 2d). Taken together, these data indicated that inflammation in synovium corresponded with ACPA-IgG autoantibodies of low avidity, reflecting an 'immature' autoimmune response. Moreover, ACPA-IgG levels in serum were not exclusively derived from plasma cells in inflamed synovium, because patients with low synovial inflammation had high circulating levels of ACPA-IgG with relatively high avidity.

### After immunoablative therapy, humoral autoimmunity is regenerated by ACPA-IgG autoantibodies of low avidity

**Figure 3:**
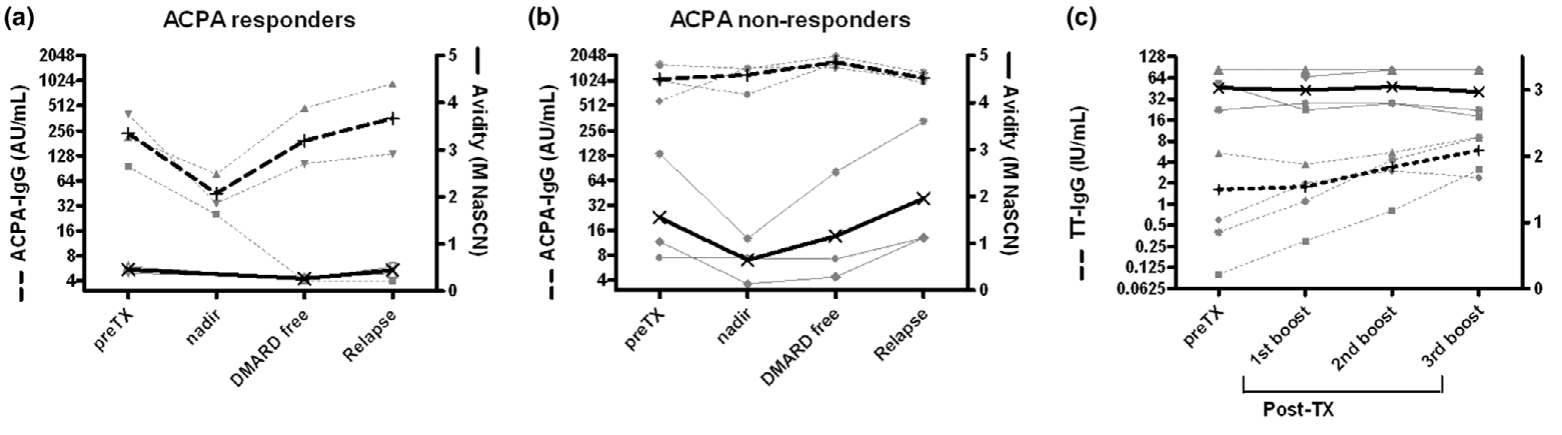
Regeneration after immunoablative therapy of ACPA-IgG autoantibodies with low avidity. **(a) **Low avidity of antibodies against anti-cyclic citrullinated protein (ACPA)-IgG (solid lines) before and after immunoablative therapy when autoimmunity is reactivated with rising levels of ACPA-IgG (dotted lines) in the three 'ACPA responders'. Levels below the detection limit were arbitrarily assigned a value of 4 arbitrary units (AU)/ml to optimize the graphical representation. Thick lines represent mean values. **(b) **Avidity of ACPA-IgG (solid lines) before and after immunoablative therapy in the three 'ACPA nonresponders', in whom, despite relatively stable ACPA-IgG levels (dotted lines), reactivation of autoimmunity was accompanied by the avidity maturation of ACPA-IgG autoantibodies. Thick lines represent mean values. **(c) **Avidity of tetanus toxoid (TT)-IgG (solid lines) before and after immunoablative therapy in four patients. Increasing levels of TT-IgG (dotted lines) after repeated TT immunizations (see Materials and methods) were accompanied by stably high avidity of TT-IgG, indicative of an intact humoral recall response despite immunoablative therapy. Thick lines represent mean values. DMARD, disease-modifying antirheumatic drug; NaSCN, sodium thiocyanate.

Clinical relapses, which occurred between 38 and 530 days after HDC plus HSCT, were preceded by a rise in ACPA-IgG (30 to 217 days) and RF-IgM levels (30 to 388 days), respectively (*P *= 0.028 and *P *= 0.043). To study the reactivation of humoral autoimmunity after immunoablative therapy, we analyzed the avidity of reappearing ACPA-IgG autoantibodies by dichotomizing patients according to their pre-transplantation ACPA-IgG level and corresponding reduction after immunoablative therapy ('ACPA responders' and 'ACPA nonresponders'; Figure 3a,b).

In the ACPA responders the decline in ACPA-IgG levels after treatment and the rise preceding clinical relapse were associated with only minor changes in the avidity of ACPA-IgG autoantibodies, which remained relatively low. However, in the ACPA nonresponders the avidity of ACPA-IgG exhibited a clear reduction (pre-transplantation median 1.04 M [range 0.69 to 2.91 M] versus median nadir after HDC plus HSCT 0.69 M [range 0.14 to 1.11 M]), reflecting low avidity ACPA-IgG autoantibodies after immunoablative therapy. Overall, the avidity of ACPA-IgG autoantibodies detectable after HDC plus HSCT in both ACPA responders and nonresponders consistently exhibited a decrease after HDC plus HSCT (median 0.27 M, range 0.14 to 1.11 M) as compared with the avidity before transplantation (median 0.69, range 0.39 to 2.91 M). Thereafter, despite relatively stable levels of ACPA-IgG toward the time of relapse of RA, avidity maturation was observed in the ACPA nonresponders, with a median of 1.12 M (range 1.12 to 3.60 M).

Collectively, these data indicate that clinically overt flares of synovitis after HDC plus HSCT were preceded by the re-emergence of low avidity ACPA-IgG autoantibodies, which is indicative of a newly generated autoimmune response. Additionally, during progression toward disease relapse after HDC plus HSCT, avidity of ACPA-IgG autoantibodies returned to pre-transplantation values.

### Humoral memory responses to recall vaccination antigen are not eradicated by immunoablative therapy

In general, humoral immunity to recall antigens depends on activation of long-lasting memory B cells and plasma cells producing high avidity antibodies. This contrasted with our findings of ACPA-IgG autoantibodies, raising the question of whether immunoablative therapy resulted in the eradication of humoral memory. Therefore, we analyzed the effects of immunoablative therapy on memory responses before and after repeated immunizations with the recall antigen TT.

As expected, the avidity of TT-IgG antibodies before transplantation was high (median 3.10 M, range 2.70 to 3.30 M). In four out of six patients, serum levels of TT-IgG increased following repeated immunizations after HDC plus HSCT, but the avidity remained high (median after first boost 3.0 M, range 2.70 to 3.30 M; after second boost 3.05 M, range 2.80 to 3.30 M; after third boost 3.0 M, range 2.60 to 3.30 M; Figure 3c). These data indicate that matured immune responses against a recall antigen remained intact despite immunoablative therapy, indicating a clear discrepancy between the humoral immunity against recall antigens and citrullinated autoantigens.

## Discussion

The aim of the present study was to investigate whether humoral autoimmune responses in six refractory RA patients, whose dysregulated immune system was ablated by HDC plus HSCT, underpinned their clinical responses. We showed that reductions in ACPA-IgG levels were associated with clinically prolonged responses to immunoablative therapy. The susceptibility of ACPA-IgG to immunoablative therapy was associated with a high degree of synovial inflammation before treatment and presence of ACPA-IgG autoantibodies of low avidity. Importantly, overt clinical relapse in all six patients was preceded by the reactivation of ACPA-IgG autoimmunity, which consisted of low avidity antibodies indicative of a newly generated ACPA-IgG response in these patients. The latter occurred despite the observation that humoral immunity against recall antigens (rubella and TT) remained unaffected by immunoablative therapy.

Previous studies have reported that avidity of autoantibodies, namely RF, anti-cardiolipin and anti-double stranded DNA autoantibodies, can also vary between patients [36,37]. The present study is the first to show that circulating ACPA-IgG autoantibodies in RA patients differ in avidity. These differences were not reflected in DAS_44 _scores, number or distribution of swollen and tender joints, or concentrations of inflammatory markers (Table 1). We did observe a striking correlation at baseline between the level of ACPA-IgG and their avidity, although this correlation between level and avidity was not observed after immunoablative therapy, indicating that this finding was probably serendipidous. More importantly, we demonstrated that in one group of patients (patients 1 to 3; ACPA responders) the presence of low avidity ACPA-IgG autoantibodies was associated with a high degree of synovial inflammation, suggesting that production of low avidity ACPA-IgG was derived from plasma cells in inflamed synovium. This was further supported by the finding that ACPA-IgG levels correlated well with C-reactive protein levels in these patients and were effectively reduced by immunoablative therapy. In contrast, clinical nonresponders had high serum levels of ACPA-IgG with relatively high avidity and a low degree of synovial inflammation, suggesting that ACPA-IgG in these patients (patients 4 to 6) was mainly produced by plasma cells from bone marrow or other secondary lymphoid organs. Together, these data suggest that ACPA-IgG levels in serum reflect production at different sites (for example, inflamed synovium or bone marrow). Further studies are needed to investigate whether ACPA-IgG autoantibodies of high avidity are indeed produced by bone marrow derived plasma cells or whether other mechanisms are responsible for the differences in avidity of ACPA autoantibodies, for instance consumption of high avidity ACPA-IgG autoantibodies at sites of synovial inflammation.

By exploiting the antiproliferative and immunoablative properties of HDC plus HSCT, the present study was able to identify two different phenotypes of refractory RA patients on the basis of the level and avidity of circulating ACPA-IgG autoantibodies. As a consequence, high serum levels of ACPA-IgG with high avidity in RA patients can account for the seemingly paradoxical reports that high serum levels of ACPA-IgG predicted unresponsiveness to TNF-blocking therapy [17] and, at the same time, that decreases in ACPA-IgG levels are associated with response to TNF-blocking therapy [16,38]. It is tempting to speculate that the presence of high avidity ACPA-IgG autoantibodies is characteristic of a category of RA patients who are refractory or less responsive to immunosuppressive treatment. In view of this hypothesis, it is noteworthy that one patient, with low avidity ACPA-IgG autoantibodies before transplantation, exhibited a long-lasting absence of detectable ACPA-IgG levels after HDC plus HSCT. One previous study [39] reported eradication of serum ACPA-IgG in eight patients with early RA receiving conventional antirheumatic treatment. To our knowledge, however, the present study is the first to show eradication of ACPA-IgG autoantibodies in refractory, persistent RA (disease duration 7 years) that simultaneously resulted in complete remission (lowest DAS_44 _score 0.89), with discontinuation of antirheumatic treatment for more than 1.5 years.

Another important observation was the discrepancy between the humoral immune responses against recall antigens and citrullinated autoantigens. The present study convincingly demonstrated that the ability to mount a recall response against TT remained intact after immunoablative therapy. The latter was not the case for the response to citrullinated antigens, as indicated by the nearly complete eradication of ACPA-IgG autoantibodies in the ACPA responder group after HDC plus HSCT, and by the decrease in ACPA-IgG avidity in the ACPA nonresponder group, despite relatively stable levels of serum ACPA-IgG. The latter strongly suggested that in the ACPA nonresponder group, during the short DMARD-free period after transplantation, a relapse of RA-related synovial inflammation was heralded by a newly generated ACPA immune response. However, our data could not discriminate between ablation by HDC plus HSCT of autoreactive memory in the ACPA nonresponder group and nonexistence of autoreactive memory B cells.

## Conclusion

This study demonstrated an association in individual RA patients between clinical response and susceptibility of ACPA-IgG autoantibodies to immunoablative therapy. Response to HDC plus HSCT was associated with the presence of low avidity ACPA-IgG autoantibodies and histological features of active synovitis. Additionally, clinical relapse after HDC plus HSCT was preceded by the emergence of low avidity ACPA-IgG autoantibodies, indicative of a newly generated autoimmune response during synovial inflammation. These data give novel insight into the humoral autoimmune response in RA.

## Abbreviations

ACPA = anti-cyclic citrullinated protein antibody; AU = arbitrary units; DAS_44 _= Disease Activity Score for 44 joints; DMARD = disease-modifying antirheumatic drug; ELISA = enzyme-linked immunosorbent assay; HDC = high-dose chemotherapy; HSCT = haematopoietic stem cell transplantation; NaSCN = sodium thiocyanate; PC-IgM = anti-phosphorylcholine of the IgM isotype; RA = rheumatoid arthritis; RF = rheumatoid factor; RL-IgG = rubella of the IgG isotype; TNF = tumour necrosis factor; TT = tetanus toxoid.

## Competing interests

The authors declare that they have no competing interests.

## Authors' contributions

YKOT contributed to the acquisition of data, analysis and interpretation of data, and preparation of the manuscript. RJV contributed to the design of the study and acquisition of the data. KNV contributed to the acquisition of data. GWND contributed to the acquisition of data. IMB contributed to the acquisition and analysis of data. MJDvT contributed to the interpretation of data. CMJ-vZ contributed to the acquisition, analysis and interpretation of the data. REMT contributed to the interpretation of the data. TWJH contributed to interpretation of data. JMvL contributed to the design of the study, acquisition, analysis and interpretation of the data, and preparation of the manuscript.
